# Disequilibrium of Blood Coagulation and Fibrinolytic System in Patients With Coronary Artery Ectasia

**DOI:** 10.1097/MD.0000000000002779

**Published:** 2016-03-03

**Authors:** Wei Wu, Ruifeng Liu, Lianfeng Chen, Houzao Chen, Shuyang Zhang

**Affiliations:** From the Department of Cardiology, Chinese Academy of Medical Sciences and Peking Union Medical College, Peking Union Medical College Hospital (WW, RL, LC, SZ); and National Laboratory of Medical Molecular Biology, Institute of Basic Medical Sciences, Chinese Academy of Medical Sciences and Peking Union Medical College (HC), Beijing, China.

## Abstract

Thrombus formation and myocardial infarction are not uncommon in patients with coronary artery ectasia (CAE). In light of this, the present study aims to systemically evaluate the blood coagulation and fibrinolytic systems in CAE patients. In this study, we enrolled 30 patients with CAE, 30 patients with coronary atherosclerosis disease (CAD), and 29 subjects with normal coronary arteries (control). The coagulation system was evaluated using a routine coagulation function test performed in the hospital laboratory before coronary angiography, and measurements included prothrombin time, international normalized ratio, activated partial thromboplastin time, fibrinogen time, and thrombin time. The evaluation of the fibrinolytic system included measurements of D-dimer, euglobulin lysis time, plasminogen activator inhibitor 1, plasminogen, plasminogen activity assay, α1-antitrypsin (α1-AT), α2 plasmin inhibitor (α2-PI), and α2-macroglobulin (α2-MG). Alpha1-AT, α2-PI, and α2-MG also inhibit activities of 3 neutrophil serine proteases, namely human neutrophil elastase (HNE), cathepsin G (CG), and proteinase 3 (PR3); therefore, the plasma levels of these 3 proteinases were also evaluated.

In CAE patients, the circulating coagulation system was normal. For the fibrinolytic system, a decrease of plasminogen activity was observed (*P* = 0.029) when compared with CAD patients, and the concentrations of α1-AT (both *P* < 0.001), α2-PI (*P* = 0.002 and *P* = 0.025), and α2-MG (*P* = 0.034 and *P* < 0.001) were significantly elevated when compared with CAD patients and normal controls. Moreover, the plasma levels of HNE (both *P* < 0.001) and CG (*P* = 0.027 and 0.016) in CAE patients were also significantly higher than those of the CAD and control groups. There was no difference in plasma PR3 concentration among these 3 groups.

Disequilibrium of the coagulation/fibrinolytic system may contribute to thrombus formation and clinical coronary events in patients with CAE. The increased plasma concentrations of α1-AT, α2-PI, and α2-MG might provide beneficial effects by inhibiting the proteinases and restraining the ectatic process; on other hand, they led to unfavorable results by inhibiting plasmin and decreasing thrombus degradation in CAE patients.

## INTRODUCTION

**FIGURE 1:**
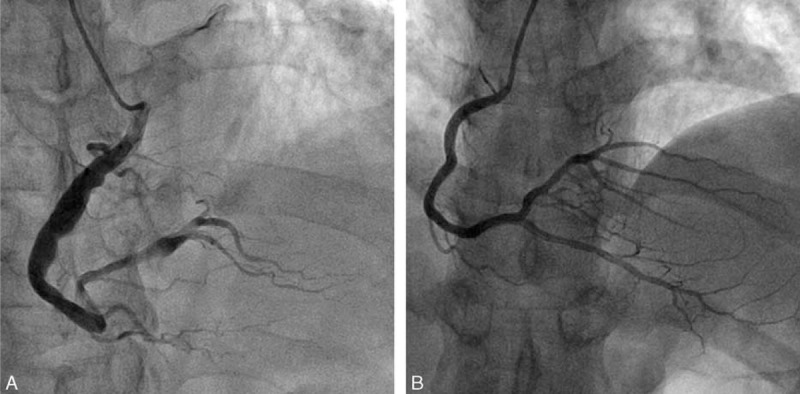
Angiography of significant ectatic right coronary artery (A) and normal right coronary artery (B).

Coronary artery ectasia (CAE) is defined as the abnormal dilatation of coronary arteries with a luminal diameter ≥1.5 times wider than that of adjacent normal segments (Figure 1).^1^ More than 50% of CAE patients had obstructive coronary artery atherosclerosis,^2^ and the right coronary artery was most frequently involved in the ectatic process.^3^ Mortality and myocardial infarction rates were higher in patients with both coronary atherosclerosis disease (CAD) and CAE than in patients with only CAD.^4^ Observational studies have demonstrated that plaque rupture and thrombosis formation at the ectatic segment are the major causes of cardiovascular events and subsequent sudden cardiac death in CAE patients.^5,6^ Acute myocardial infarction (AMI) was observed in 39.5% of CAE patients; for 58.1% patients, the ectatic segments were within the culprit vessel.^7^ In the setting of AMI, coronary ectasia had a significantly lower incidence of successful reperfusion, and massive intracoronary thrombus may be associated with an increased incidence of adverse outcomes after percutaneous coronary intervention.^6^ The reason why CAE increased the risk of thrombosis events remains unclear. The following possible mechanisms were proposed: abnormal blood flow patterns inside the coronary aneurysm were associated with thrombus formation; endothelial dysfunction; rupture of the vulnerable plaque might initiate the thrombus formation; disequilibrium of blood coagulation and the fibrinolytic system; and other possible reasons such as chronic inflammatory conditions^8^ and dysfunction of platelet aggregation. To date, there has been limited data on the topic of activities of the blood coagulation system and the fibrinolytic system in CAE patients. Global fibrinolytic capacity was found to be significantly higher in ectatic patients than in controls.^9^ However, it remains unclear whether specific changes in the coagulation/fibrinolytic system resulted in or from CAE. The objective of this study is to further investigate the process of thrombus formation and degradation in CAE patients.

The coagulation system includes 2 initial pathways that lead to fibrin formation: the contact activation pathway (also known as the intrinsic pathway), which can be evaluated by measuring activated partial thromboplastin time, and the tissue factor pathway (also known as the extrinsic pathway), which can be evaluated by measuring prothrombin time and international normalized ratio. Both pathways can trigger the activation of prothrombin, which in turn transforms fibrinogen (Fbg) into fibrin. The quantitative and qualitative evaluation of Fbg is performed by measurement of thrombin clotting time. The fibrinolytic system plays a major role in the process of thrombus degradation. In the fibrinolytic system, plasminogen (PLG) is activated into plasmin by tissue plasminogen activator and urokinase plasminogen activator, while the plasminogen activator inhibitor-1 (PAI-1) acts as the principal inhibitor of tissue plasminogen activator and urokinase plasminogen activator. Blood plasmin promotes the degradation of fibrin and thrombus; however, α1-antitrypsin (α1-AT), α2 plasmin inhibitor (α2-PI), and α2-macroglobulin (α2-MG) serve as intrinsic inhibitors of plasmin. Euglobulin lysis time is a parameter that reveals overall fibrinolysis function, and the D-dimer is the degradation product of cross-linked fibrin. This reflects ongoing activation of the hemostatic system. Importantly, α1-AT, α2-PI, and α2-MG are also inhibitors of 3 major neutrophil serine proteases, namely human neutrophil elastase (HNE), cathepsin G (CG), and proteinase 3 (PR3);^10–12^ therefore, these 3 proteases were also examined in this study.

## METHODS

### Patient Population

A total of 1239 consecutive patients who had undergone coronary angiograms from October 2013 to July 2014 at the cardiac catheterization center of our hospital were included in this study. Among these patients, 42 (3.39%) had CAE, and 30 of them who did not have conditions listed in the exclusion criteria were included in the CAE group. Thirty patients with angiographically documented CAD and 29 subjects with relatively normal coronary arteries (control) during the same time period were randomly selected. These 3 groups were matched in age, gender, and other baseline characteristics. This study was approved by the local ethics committee and was in accordance with the Declaration of Helsinki. Written informed consent was obtained from all study participants.

### Angiographic Definition

At least 2 experienced interventional cardiologists who were blinded to the patients’ original status were required to make a consentaneous diagnosis. Coronary angiography was routinely performed using the Judkins technique in multiple projections without intravenous nitroglycerin. CAE was defined as an ectatic artery diameter ≥1.5 times that of adjacent normal segments.^13^ CAD was defined as ≥50% stenosis in one or more major coronary arteries. Subjects with ≤20% coronary artery stenosis were considered normal controls.

### Exclusion Criteria

The exclusion criteria included acute coronary syndrome, cardiomyopathy, valvular heart disease, congestive heart failure, aneurysm in other vessels, hematological disorders including coagulopathies, collagen tissue diseases, vasculitis, syphilis, chronic obstructive lung disease, pulmonary hypertension, early menopause, thyroid disease, organic hepatic diseases, renal failure, known malignancy, local or systemic infection, previous history of infection (≤3 months), other inflammatory diseases, and any medications that could potentially interfere with the measurement of these markers, such as oral anticoagulations and corticosteroids.

### Medical Records and Blood Samples

Clinical data in this study were extracted from the electronic medical records of our hospital. The blood samples were collected by sodium citrate vacuum blood collection tube immediately before coronary angiography, and plasma samples were separated within 6 hours and kept at −80 °C. The coagulation and function examination was performed in the hospital laboratory as a routine test before coronary angiography, and the other experiments were performed by the members of our research team.

### Enzyme-Linked Immunosorbent Assay (ELISA)

ELISA kits for PLG, α1-AT, α2-PI, and HNE were purchased from Elabscience (Elabscience Biotechnology Co., Ltd, Wuhan, China). The kits for PR3 and CG were purchased from Cusabio (Cusabio Life Science Inc., Wuhan, China). The kits for PAI-1 and α2-MG were purchased from Boster (Boster Biological Engineering Co., Ltd, Wuhan, China). Quantification of the above targets was performed using sandwich ELISA kits following the manufacturer's instructions. The final concentrations were calculated by interpolation from standard curves.

### Euglobulin Lysis Time Detection

A total of 0.5 mL of each plasma sample was used.^14^ After the samples were prepared, 7.5 mL distilled water and 0.12 mL 1% acetic acid were added in turn. The samples were then placed in an ice bath for 10 minutes. Then, the mixture was centrifuged at 3000 rpm for 10 minutes, and the euglobulin was precipitated. The supernatant was removed, and 0.25 mL borate buffer (pH 9.0) was added to dissolve the euglobulin. Then, 0.5 mL of 0.025 M calcium chloride was added to solidify the euglobulin. The time it took the clot to completely dissolve was recorded as the euglobulin lysis time.

### Plasminogen Activity Assay

The activity of PLG was evaluated by the streptokinase-activated PLG mediated proteolysis of chromogenic substrate S-2251 (H–D-Val-Leu-Lys-pNA; Boatman Biotech Co., Ltd, Shanghai, China).^15^ In brief, the plasma samples were diluted 40 times using 0.05 M Tris-HCl buffer (pH 7.4). A total of 50 μL of each diluted sample was then incubated with 50 μL streptokinase solution (5000 IU/mL) at 37 °C for 30 minutes in a 96-well plate. Then, 50 μL S-2251 (5 g/L) was added to the sample, and the absorbance of the wells was measured at 405 nm every 30 seconds for 30 minutes. PLG activity was determined by comparing the absorbance values of the samples with that of standard human plasma.

### Statistical Analysis

Statistical analyses were performed using SPSS 17.00 package software (SPSS Inc., Chicago, IL). General descriptive characteristics are presented as the mean ± standard deviation (SD). Categorical data are presented as percentages (%). Gender and risk factors were compared using Chi-square test. Continuous numeric data were tested by one-way analysis of variance or the Kruskal–Wallis test. Before using one-way analysis of variance, the normality test (Shapiro–Wilk test) and Levene test have to be finished, and the Kruskal–Wallis test should be employed while the data were in a nonnormal distribution. The LSD method (normal distribution) or the Nemenyi test (nonnormal distribution) was used for multiple comparisons between the groups. The lowest level of significance accepted was *P* < 0.10.

## RESULTS

### Basic Clinical Characteristics

The baseline characteristics of the CAE, CAD, and control groups are described in Table 1. The 3 groups were similar with regards to age, gender, presence of hypertension, blood pressure, fasting glucose, lifestyle, lipid profile, and other traditional cardiovascular risk factors, with the exception of having a family history of CAD. The blood cell subtype counts, hs-CRP, hepatic, and renal functions were also comparable among the 3 groups. The right coronary artery and left circumflex were the most frequently affected arteries, and the left anterior descending and left main coronary artery exhibited much lower incidences of ectasia in CAE patients. These results are similar to previous findings.

**TABLE 1:**
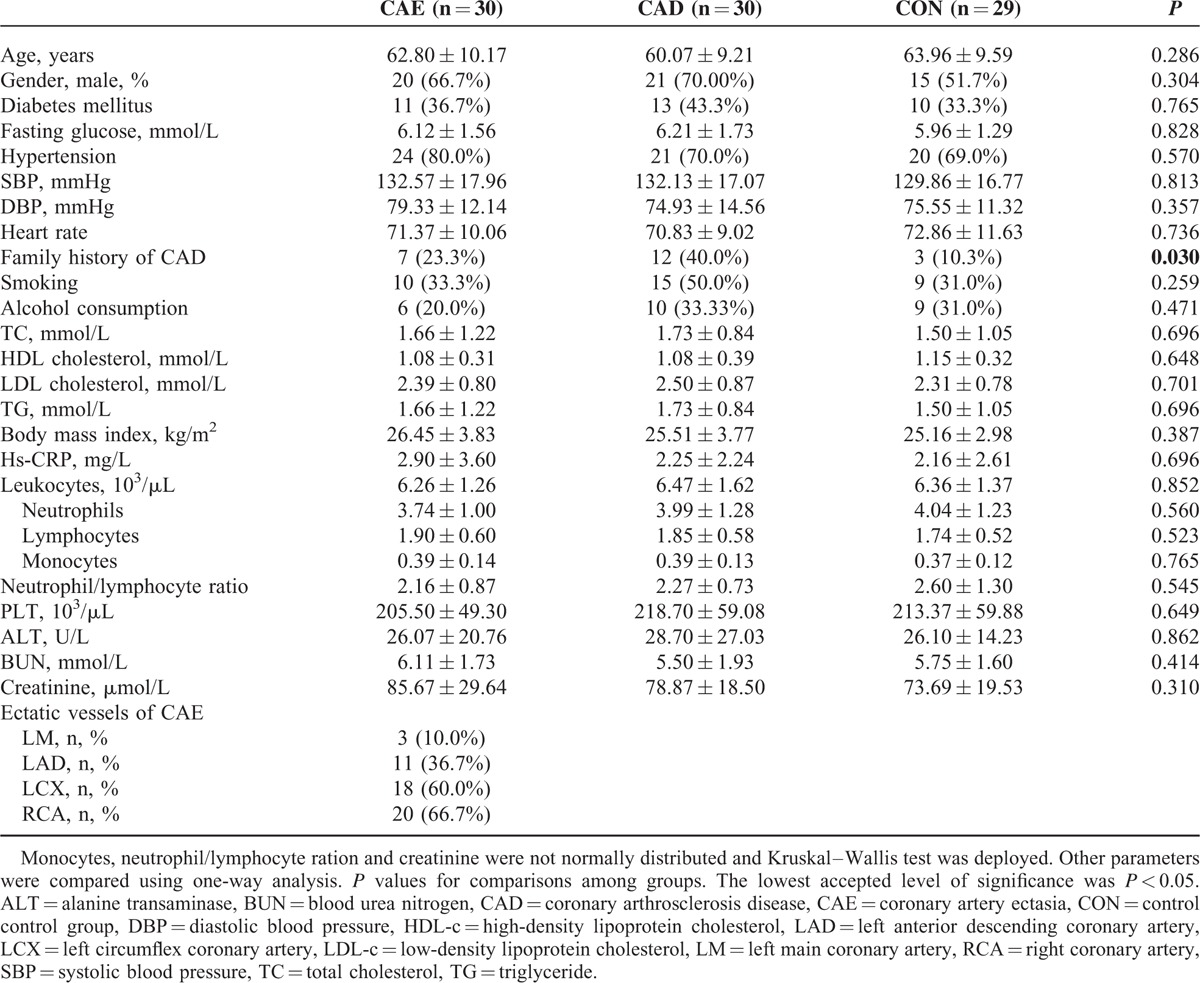
Clinical Characteristics of the Enrolled Subjects

### The Coagulation System in CAE Patients

As shown in Table 2, all routine indexes of coagulation function including prothrombin time, international normalized ratio, activated partial thromboplastin time, Fbg, and thrombin clotting time were comparable among the CAE, CAD, and control groups. There were no significant differences in coagulation function between the groups.

**TABLE 2:**
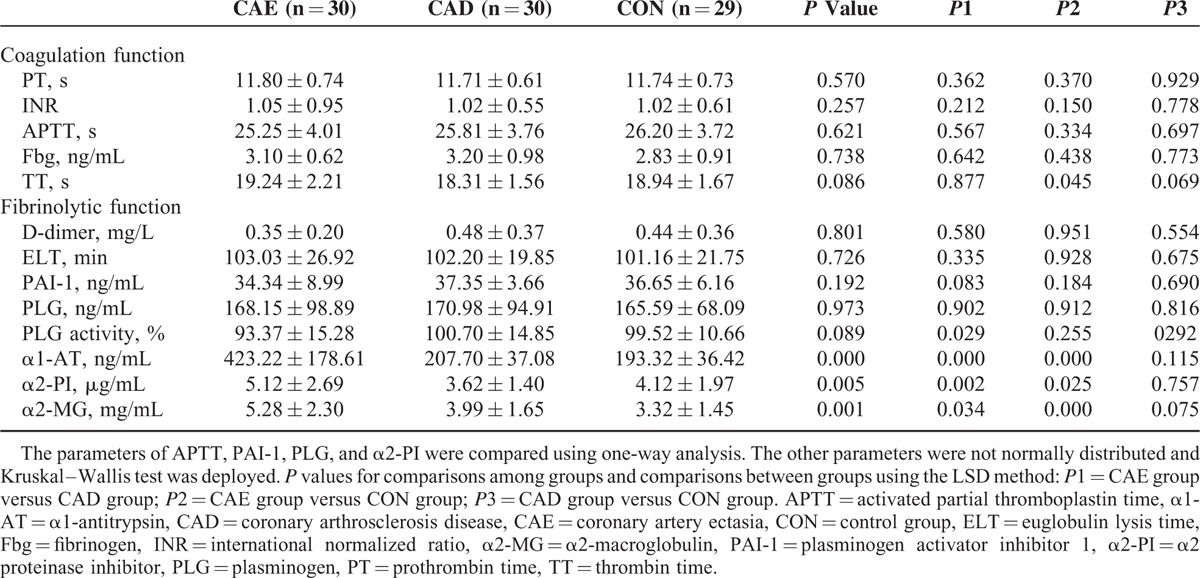
The Preliminary Evaluation of the Blood Coagulation and Fibrinolytic Systems

### The Fibrinolytic System in CAE Patients

Similar levels of the D-dimer, euglobulin lysis time, PAI-1, and PLG were observed among the CAE, CAD, and control groups (Table 2). However, the PLG activity in CAE patients was significantly lower than that of the CAD (*P* = 0.029). The plasma levels of α1-AT, α2-PI, and α2-MG in the CAE group were significantly higher than those of the CAD group (*P* < 0.001, *P* = 0.002, and *P* = 0.034, respectively) and the control group (*P* < 0.001, *P* = 0.025, and *P* < 0.001, respectively) (Table 2).

### Circulating Neutrophil Serine Proteases in CAE Patients

As shown in Table 3, both the concentrations of HNE and CG were significantly higher in the CAE group than in the CAD (*P* < 0.001 and *P* = 0.027, respectively) and control groups (*P* < 0.001 and *P* = 0.016, respectively). However, there was no significant difference in the level of PR3 among the 3 groups (*P* = 0.376).

**TABLE 3:**
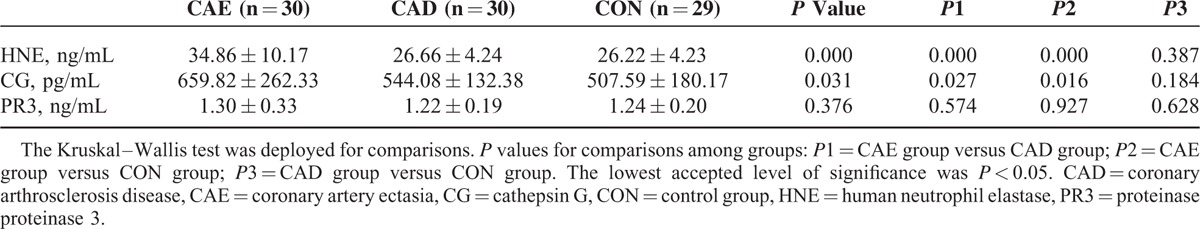
Comparison of the Concentrations of 3 Neutrophil Serine Proteases Among the Groups

## DISCUSSION

This study mainly evaluated the coagulation system and the fibrinolytic system in patients with ectatic coronary arteries. Our results showed that the coagulation system in CAE patients was normal, while the fibrinolytic system was partly abnormal, which might have an effect on the process of thrombus degradation. Such disequilibrium in the coagulation and fibrinolytic systems may increase the risk of myocardial infarction and other coronary thrombotic events in CAE patients. Because most CAE patients had obstructive coronary artery disease (80% of the CAE patients in this study had at least 1 vessel stenosis >50% simultaneously), the CAD group was enrolled to account for the effect of atherosclerosis. The baseline clinical characteristics were comparable among the 3 groups with the exception of a family history of CAD.

Comparable circulating coagulation function among the CAE, CAD, and control groups did not eliminate the possibility that thrombus formation may be improperly triggered. In fact, in the ectatic vessels and/or segments of CAE patients, the presence of slow and turbulent blood flow,^16–18^ endothelial dysfunction,^19,20^ vulnerable plaque,^21^ proatherogenic and prothrombotic properties of lipoprotein(a),^22^ chronic inflammatory conditions^16,23–25^ as well as platelet dysfunction^26,27^ can facilitate thrombus formation. However, most of those factors could not be evaluated by in vitro tests, or in some cases the plasma levels of cytokines and/or biomarkers were not precisely consistent with the changes in the endothelium and vascular wall of the localized coronary arteries. Our study did not exclude the possibility of hypercoagulation in local ectatic arteries.

After the process of thrombus formation was initiated in a local segment, theoretically, under normal conditions, the fibrinolytic system would be activated immediately and degradation of the thrombus would ensue. The major abnormal results in this study are the circulating changes of the fibrinolytic system. Decreased levels of PLG activity and increased concentrations of plasmin inhibitors (α1-AT, α2-PI, and α2-MG) were identified in CAE patients. Although the PLG concentration was normal in patients with CAE, the PLG activity was decreased by 6.2% and 7.2%, respectively, compared with normal controls and patients with CAD. Increased concentrations of plasmin inhibitors could be partially responsible for the observed changes. With the disequilibrium of the coagulation and fibrinolytic systems, the fibrinolytic process might not be effectively activated to antagonize thrombus formation, which was triggered by the previously mentioned mechanisms. Under this condition, the newly developed thrombus would not be sufficiently and rapidly enzymolyzed by plasmin, and the subsequent cascade of thrombus formation and platelet aggregation would eventually result in coronary thrombotic events. Similar to the coagulation status, the localized fibrinolytic activity in the ectatic area may differ from the fibrinolytic function at the circulation level.

Increased levels of α1-AT and α2-MG in CAE patients were first reported in 1988, when researchers suggested that coronary ectasia was associated with disturbances in the protease-antiprotease system.^28^ However, no subsequent studies were ever conducted to investigate the underlying mechanism. In this study, focusing on coagulation and fibrinolysis, we once again verified the increase of α1-AT and α2-MG, and also demonstrated an increase of α2-PI in patients with CAE compared with CAD patients and normal controls. It is worth noting that these 3 plasmin inhibitors are also inhibitors of numerous proteinases. α2-PI is an irreversible inhibitor of HNE, PR3, CG, and other proteinases.^11,29,30^ α1-AT is the primary fast-acting inhibitor of plasmin in vivo but has also been reported to inhibit many enzymes such as trypsin, elastase, and activated protein C.^31^ α2-MG is a polyvalent homotetrameric inhibitor, which inhibits a substantial classes of proteases.^11,29,30^ As reported in one of our previous studies,^32^ the proteolytic enzymes may play vital roles in the process of extracellular destruction because they usually serve as terminal effectors mediating tissue destruction. Thus, we hypothesize that increases in the 3 plasmin inhibitors might function to consecutively restrict the related proteinases of leukocytes. In this study, the increased plasma concentrations of HNE and CG in the CAE group provided evidence for our hypothesis. The circulating PR3 level did not change in the CAE group. One possible explanation is that PR3 is stored mainly in azurophilic granules or is constitutively expressed on the membranes of neutrophils.^33^

Characteristic pathological manifestations of CAE include extensive destruction of musculoelastic elements; and elastin fibers are the dominant components of the extracellular matrix of the coronary artery wall.^13,34^ In our previous study, circulating soluble elastin was significantly higher in CAE patients than in the CAD and control groups, which indicated the degradation of elastin fibers as one of the main changes in extracellular matrix metabolism.^32^ Considering the fact that elastin fibers are one of the main substrates of HNE, PR3, and CG,^10,35,36^ we hypothesize that the elevated concentrations of these neutrophil serine proteases might be the underlying mechanism for CAE, and the levels of their inhibitors are correspondingly increased to restrict their activity. At the same time, those inhibitors, such as α1-AT, α2-PI, and α2-MG, had other functions including the inhibition of the activity of plasmin, which may lead to the disequilibrium of the coagulation/fibrinolytic system in CAE patients.

## CONCLUSIONS

In CAE patients, the circulation coagulation system is normal, while the fibrinolytic system is partially restrained. In the fibrinolytic system, the PLG activity is inhibited possibly by elevated plasma levels of α1-AT, α2-PI, and α2-MG, which also function as inhibitors of the neutrophil serine proteases related to the pathological process of coronary ectasia. In other words, the increased plasma concentrations of α1-AT, α2-PI, and α2-MG might provide beneficial effects by inhibiting the proteinases and restraining the ectatic process. On the other hand, they may lead to unfavorable results by inhibiting plasmin and decreasing thrombus degradation in CAE. Our results may provide a better understanding as to why patients with CAE are more prone to having a higher thrombus burden in the setting of AMI and to developing no-reflow during percutaneous coronary intervention.

## LIMITATION

All experimental data were collected from the analyses of peripheral blood samples. The changes in the circulating markers may not always match the changes in the localized ectatic coronary arteries. However, CAE was considered a chronic systemic disease; because the 3 groups had distinct features in the coronary vascular walls, the circulating markers should reflect the transformation in ectatic coronary arteries, at least to some degree. Based on structural and functional changes, although we have no evidence, it is possible that there is higher coagulation function and lower fibrinolytic function in localized ectatic coronary arteries than in the peripheral blood.
